# A Phosphorylated Dendrimer-Supported Biomass-Derived Magnetic Nanoparticle Adsorbent for Efficient Uranium Removal

**DOI:** 10.3390/nano14090810

**Published:** 2024-05-06

**Authors:** Mingyang Ma, Qunyin Luo, Ruidong Han, Hongyi Wang, Junjie Yang, Chunyuan Liu

**Affiliations:** State Key Laboratory of Nuclear Resources and Environment, East China University of Technology, Nanchang 330013, China

**Keywords:** uranium adsorption, magnetic nanoparticles, chitosan, poly(amidoamine), adsorbent regeneration, adsorption mechanism

## Abstract

A novel biomass-based magnetic nanoparticle (Fe_3_O_4_-P-CMC/PAMAM) was synthesized by crosslinking carboxymethyl chitosan (CMC) and poly(amidoamine) (PAMAM), followed by phosphorylation with the incorporation of magnetic ferric oxide nanoparticles. The characterization results verified the successful functionalization and structural integrity of the adsorbents with a surface area of *ca*. 43 m^2^/g. Batch adsorption experiments revealed that the adsorbent exhibited a maximum adsorption capacity of 1513.47 mg·g^−1^ for U(VI) at pH 5.5 and 298.15 K, with Fe_3_O_4_-P-CMC/G1.5-2 showing the highest affinity among the series. The adsorption kinetics adhered to a pseudo-second-order model (*R*^2^ = 0.99, *q*_e,exp_ = 463.81 mg·g^−1^, *k*_2_ = 2.15×10^−2^ g·mg^−1^·min^−1^), indicating a chemically driven process. Thermodynamic analysis suggested that the adsorption was endothermic and spontaneous (ΔH° = 14.71 kJ·mol^−1^, ΔG° = −50.63 kJ·mol^−1^, 298. 15 K), with increasing adsorption capacity at higher temperatures. The adsorbent demonstrated significant selectivity for U(VI) in the presence of competing cations, with Fe_3_O_4_-P-CMC/G1.5-2 showing a high selectivity coefficient. The performed desorption and reusability tests indicated that the adsorbent could be effectively regenerated using 1M HCl, maintaining its adsorption capacity after five cycles. XPS analysis highlighted the role of phosphonate and amino groups in the complexation with uranyl ions, and validated the existence of bimodal U4f peaks at 380.1 eV and 390.1 eV belonging to U 4f_7/2_ and U 4f_5/2_. The results of this study underscore the promise of the developed adsorbent as an effective and selective material for the treatment of uranium-contaminated wastewater.

## 1. Introduction 

Although nuclear power presents itself as a sustainable alternative to fossil fuels, offering reduced emission of greenhouse gases [1], the challenge of managing environmental legacy looms large due to the production of chemically and radioactively toxic waste throughout the nuclear fuel cycle [2]. Uranium, a critical component of nuclear reactors [3], is the primary source of radioactive waste, posing significant risks, as it can contribute to background radiation and potentially induce genetic mutations in living organisms if it is released into the environment [4]. Consequently, there is a growing dedication to research aimed at mitigating the environmental impact associated with the treatment of wastewater from the fuel cycle [5]. To date, a variety of physical [6] and chemical [7] techniques have been suggested for the extraction of uranium from contaminated water sources, with adsorption emerging as a preferred method due to its simplicity, effectiveness, and broad application [7]. The search for superior materials that can effectively remove uranium from wastewater is ongoing.

Various techniques have been employed for the adsorption of uranium, including ion exchange [8], precipitation [9], and adsorption using natural and synthetic materials [10]. Among these, adsorption is favored for its simplicity, cost-effectiveness, and adaptability to different conditions. It is significant to highlight the exceptional benefits of magnetic nanoparticles when it comes to enhancing efficiency and retrieving suspended solids in real-world scenarios. Numerous studies have proposed that the use of magnetic nanoparticles can eliminate the need for particle filtration processes [11]. A wide range of magnetic nanoparticle adsorbents, from activated carbons to metal–organic frameworks (MOFs), have been explored for their uranium removal capabilities [12,13]. However, the challenge lies in enhancing both the adsorption capacity and selectivity of these materials, especially in the presence of competing ions, often found in nuclear wastewater. Consequently, they have garnered considerable interest among the scientific community due to their potential for adsorbing radioactive isotopes and heavy-metal ions. 

Natural biopolymers, such as cellulose [14], alginate [15], and chitosan [16], have led to applications in the removal of radionuclides from wastewater due to the higher affinity of amino, hydroxyl, and carboxyl groups towards uranyl ions. Chitosan, which is derived from chitin, is emerging in the adsorption of uranyl ions, ascribed to the coordination and electrostatic interaction sites [17]. However, the adsorption efficacy is limited because the solubility of this biopolymer depends mainly on the pH value of the solution. Carboxymethyl chitosan (CMC), an anionic derivative of chitosan, guarantees better solubility regardless of pH values as a means to render an adsorbent with tunable properties. Dendrimers that radiate in a nearly perfect hyperbranched topology structure from a central core are highly monodisperse polymers and architecturally grown generation by generation, resulting in outermost functional groups linked by interior branches [18]. Poly(amidoamine) (PAMAM) dendrimers, the first dendrimer commercialized and explored most extensively, have found applications in drug delivery [19], gene therapy [20], and environmental pollution treatment [21]. PAMAM dendrimers stand out as a potential candidate for the treatment of radioactive wastewater due to the diverse possibilities of modification based on the NH_3_ or COOCH_3_ terminal characteristics and their excellent hydrophilic molecular building blocks [22,23]. However, few studies have been conducted regarding the utilization of PAMAM for the adsorption of uranyl ions.

In this study, magnetic nanoparticle composites with dual hydrophilic compounds, CMC and phosphorylated PAMAM (Fe_3_O_4_-P-CMC/PAMAM), were prepared for the first time. Diverse methods were employed to analyze the surface characteristics, structure, and porosity of Fe_3_O_4_-P-CMC/PAMAM, including SEM, FT-IR, BET, Zeta potential, and XRD. The effects of the pH value of the solution, the solution temperature, and the coexisting competing ions were thoroughly examined. Systematical studies investigated the adsorption efficacy of the as-prepared adsorbent, evaluating its capacity, selectivity, and reusability. Additionally, the bonding dynamics between the phosphonate groups and uranyl ions were inferred by examinations conducted by XPS.

## 2. Experimental 

### 2.1. Reagents

Carboxymethyl chitosan (CMC) was purchased from Shanghai Macklin Biochemical Co., Ltd. (Shanghai, China), while ethylenediamine, methyl acrylate, FeCl_3_·6H_2_O, FeCl_2_·4H_2_O, and other reagents were of analytical grade and were obtained from Adamas-beta. For the preparation of the uranium (VI) stock solution, 1.1792 g of U_3_O_8_ was placed in a 100 mL beaker, followed by the addition of 10 mL of hydrochloric acid (12 mol·L^−1^, ρ = 1.18 g∙mL^−1^) and 2 mL of 30% hydrogen peroxide. The mixture was then heated until nearly dry, and another 10 mL of hydrochloric acid was added. Subsequently, the solution was diluted with distilled water to a total volume of 1 L, resulting in a uranium (VI) stock solution with a concentration of 1 mg∙L^−1^.

### 2.2. Preparation of PAMAM Dendrimer (G1.5)

**Scheme 1:**
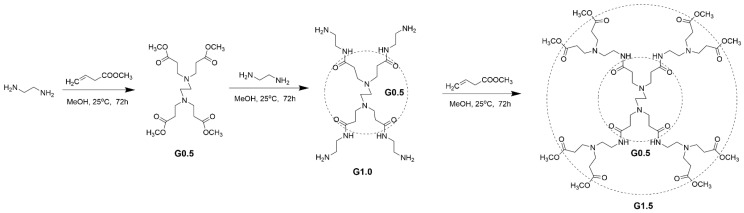
Synthetic route to the PAMAM dendrimer.

Target PAMAM dendrimers were obtained by a sequential reaction of Michael addition and amidation. In an ice water bath, 12.5 mL of methanol solution containing 21 mL of methyl acrylate was added dropwise to another 12.5 mL of methanol mixture containing 3.75 mL of ethylenediamine for 0.5 h. The mixture was stirred for 72 h at room temperature. After the reaction, all solvents were removed using a rotary evaporator to give G0.5. In an ice water bath, 10 g of G0.5 was dissolved in 12.5 mL of methanol, and another 12.5 mL of methanol containing 6.678 mL of ethylenediamine was added and stirred for 72 h at room temperature to yield G1.0. The next two synthesis steps were performed in the same order and under the same conditions as the previous steps, until highly productive target dendrimers were obtained labeled G0.5 and G1.5, respectively (Scheme 1). 

G0.5: ^1^H NMR (CDCl_3_): δ = 3.67 ppm (s, 12H, -COOCH_3_), 2.79–2.75 ppm (t, 8H, -NCH_2_CH_2_CO), 2.50 ppm (s, 4H, -NCH_2_CH_2_N), 2.46–2.42 ppm (t, 8H, -CH_2_COO). 

G1.5: ^1^H NMR (CDCl_3_): δ = 3.67 ppm (s, 24H, -COOCH_3_), 3.32–3.27 ppm (m, 8H, -CONHCH_2_), 2.82–2.73 ppm (t, 16H, -NCH_2_CH_2_CO), 2.56–2.53 ppm (t, 16H, -CH_2_N), 2.50 ppm (s, 4H, -NCH_2_CH_2_N), 2.47–2.42 ppm (t, 16H, -CH_2_COO), 2.36–2.32 ppm (m, 8H, CH_2_CONH). 

### 2.3. Preparation of CMC/G1.5 Nanoparticles

The G1.5 dendrimer at a dosage of 50, 100, and 150 mg was separately dissolved in 40 mL of methanol solution, and 100 mg of CMC was dissolved in 10 mL of water. After the G1.5 dendrimer solutions were mixed with the CMC solutions, the resultant solutions were individually diluted with 30 mL of methanol and stirred at room temperature for 72 h. After adding an appropriate volume of saturated sodium carbonate solution and acetone, white CMC/G1.5-1, CMC/G1.5-2, and CMC/G1.5-3 nanoparticles were precipitated, then aspirated and washed with deionized water and ethanol, respectively. 

### 2.4. Preparation of P-CMC/G1.5 Nanoparticles

The CMC/G1.5 series nanoparticles with a mass of 0.4 g and 30 mL of DMF with 4.0 g of urea were mixed and heated at 100 °C for 1 h, then 1.8 mL of H_3_PO_4_ was added to the above solution successively, which was heated to 135 °C and refluxed for another 6 hrs. Finally, the resulting products (P-CMC/G1.5) were washed with water and ethanol, respectively.

### 2.5. Preparation of Magnetic Fe_3_O_4_ Nanoparticles

A solution of 0.3 mol/L FeCl_3_ and FeCl_2_ was prepared in a 2:1 molar ratio, and the pH of the mixture was adjusted to 10 with ammonia, at which point the solution was stained from orange red to black. The mixture was placed at 80 °C and the reaction was executed for 0.5 h. The mixture was subsequentially washed several times with deionized water and ethanol and dried in an oven at 70 °C for 24 h. 

### 2.6. Preparation of Fe_3_O_4_-P-CMC/G1.5 Nanoparticles

**Scheme 2:**
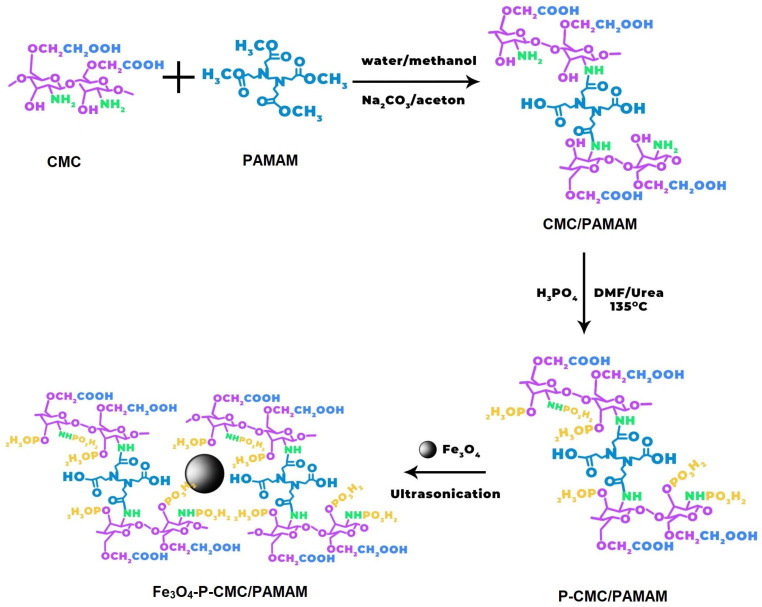
Synthetic route to magnetic Fe_3_O_4_-P-CMC/PAMAM nanoparticles.

The P-CMC/G1.5 and CMC/G1.5 nanoparticles with a mass of 0.4 g were individually dispersed in 20 mL of deionized water and sonicated for 20 min, then 5 mL of 1-(3-dimethylaminopropyl)-3-ethylcarbodiimide hydrochloride (25 mg/mL) was added to the dispersion of magnetic Fe_3_O_4_ nanoparticles, and the mixture was resonicated for 20 min. P-CMC/G1.5 and CMC/G1.5 nanoparticles were added to the magnetic particle dispersion and further sonicated for 1 h. The product was washed with deionized water and ethanol, respectively. The washed materials were then freeze-dried to obtain the magnetic nanomaterials, Fe_3_O_4_-P-CMC/G1.5 and Fe_3_O_4_-CMC/G1.5, the latter of which was selected as the control specimen (Scheme 2).

### 2.7. Effect of Different Factors on U(VI) Adsorption

The adsorption behaviors of Fe_3_O_4_-CMC/G1.5 and Fe_3_O_4_-P-CMC/G1.5 towards U(VI) were studied through batch experiments. Typically, 5.0 mg of the adsorbent was added to Erlenmeyer flasks containing U(VI) solutions with designed concentrations and pH values (adjusted using 0.1 M or 0.01 M HNO_3_ and NaOH solutions). After shaking at a specific temperature (*T*, K) for a certain duration (*t*, min), the solutions were centrifuged, and then the concentration of U(VI) in the supernatant was analyzed using the Arsenazo III method at 650 nm with a visible spectrophotometer [24]. The adsorption capacity (*q*_e_, mg·g^−1^) and distribution coefficient (*K*_d_, mL∙g^−1^) were calculated using Equations (1) and (2).

(1) $$q_{e}=\frac{(C_{0}-C_{e})\times V}{m}$$

(2) $$K_{d}=\frac{(C_{0}-C_{e})\times V}{C_{e}\times m}$$

where *C*_0_ and *C*_e_ represent the initial and equilibrium concentrations of the metal ion (mg∙L^−1^), respectively. *V* stands for the volume of the solution (L), and *m* denotes the amount of the sorbent (g).

All batch adsorption tests were performed in triplicate, and the variability in the adsorption capacity (*q*_e_) was determined by computing the average and standard deviation of the collected data points. Error bars representing this uncertainty were included in the graphical representations.

The uranium selectivity (*S*_U_) characterizes the adsorption capacity and degree of selectivity of the sorbent towards uranium [25], which can be calculated using Equation (3).

(3) $$S_{U}=\frac{q_{e(U)}}{q_{e(\mathrm{total})}}\times 100\%$$

where *q*_e(U)_ represents the adsorption capacity of uranium (mg∙g^−1^), and *q*_e(total)_ stands for the total amount of adsorption for all multi-ions by Fe_3_O_4_-CMC/G1.5 and Fe_3_O_4_-P-CMC/G1.5 (mg∙g^−1^).

Furthermore, the selectivity coefficient of uranyl ions relative to competing ions (*S*_U(VI)/M(x)_) can be studied using Equation (4):

(4) $$S_{U(\mathrm{VI})/M(x)}=\frac{K_{d}^{U(\mathrm{VI})}}{K_{d}^{M(x)}}$$

where $K_{d}^{U(\mathrm{VI})}$ and $K_{d}^{M(x)}$ represent the distribution coefficients of uranyl ion and other ions, respectively.

The relative selectivity coefficient (*S*_r_) is calculated based on Equation (5):

(5) $$S_{r}=\frac{S_{{\mathrm{Fe}}_{3}O_{4}-P-\mathrm{CMC}-G1.5-2}}{S_{{\mathrm{Fe}}_{3}O_{4}-\mathrm{CMC}-G1.5}}$$

### 2.8. Adsorption Kinetic

The linear forms of the pseudo-first-order kinetic model, pseudo-second-order kinetic model, and intraparticle diffusion model [26] are given by Equations (6)–(8), respectively.

(6) $$\ln(q_{e}-q_{t})=\ln q_{e}-k_{1}t$$

(7) $$\frac{t}{q_{t}}=\frac{1}{k_{2}q_{e}^{2}}+\frac{t}{q_{e}}$$

(8) $$q_{t}=k_{int}t^{0.5}+C$$

where *t* (min), *q*_t_, and *q*_e_ (mg·g^−1^) refer to the adsorption time, U(VI) adsorption capacity at *t,* and adsorption capacity at equilibrium, respectively; *k*_1_ (min^−1^), *k*_2_ (g∙mg^−1^∙min^−1^), and *k*_int_ (mg·g^−1^·min^−0.5)^ are the rate constants of pseudo-first-order, pseudo-second-order, and intraparticle diffusion kinetics models, respectively; and *C* (mg·g^−1^) is the constant proportional to the extent of the boundary layer thickness.

### 2.9. Adsorption Isotherms

At 298.15 K, the relationship between the equilibrium concentration of U(VI) and its adsorption capacity is often explored using three adsorption isotherm models: Langmuir, Freundlich, and Dubinin–Radushkevich (D-R). These models provide insights into the interaction mechanisms of composite materials in U(VI) solutions [27,28,29].

The Langmuir isotherm assumes monolayer adsorption on a uniform surface without interactions. The Freundlich isotherm proposes multilayer adsorption with unevenly distributed active sites. The D-R isotherm introduces the theory of pore adsorption. Furthermore, these three models are represented by Equations (9)–(11).

(9) $$q_{e}=\frac{q_{m}K_{L}C_{e}}{1+K_{L}C_{e}}$$

(10) $$q_{e}=K_{F}\times C_{e}^{1/n}$$

(11) $$lnq_{e}=lnq_{\mathrm{DR}}-\beta \varepsilon^{2}$$

where *q*_e_ and *q*_DR_ represent the amount of adsorption at equilibrium (mg∙g^−1^) and the theoretical adsorption capacity (mol·g^−1^), respectively. *q*_m_ stands for the maximum or saturated amount of adsorption (mg∙g^−1^), while *C*_e_ denotes the equilibrium concentration (mg∙L^−1^). *K*_L_ is an equilibrium constant related to the binding strength (L∙mg^−1^). *n* and *K*_F_ are Freundlich constants, indicating adsorption capacity and adsorption intensity, respectively. *β* is a constant associated with the adsorption energy (mol^2^·kJ^−2^), and *ε* represents the Polanyi potential, calculated as *ε* = R*T*ln(1 + 1/*C*_e_). R is the gas constant (kJ·mol^−1^ K^−1^), and *T* represents the absolute temperature (K).

The mean free energy of adsorption refers to the change in free energy when one mole of ions is transferred from infinity in the solution to the surface of the adsorbent [30], and its formula is represented in Equation (12).

(12) $$E_{\mathrm{DR}}=\frac{1}{{\left(-2\beta \right)}^{0.5}}$$

The magnitude of *E*_DR_ is related to the reaction mechanism. If *E*_DR_ falls within the range of 8–16 kJ·mol^−1^, the adsorption process is controlled by chemical adsorption [31]. In the case of *E*_DR_ being less than 8.0 kJ·mol^−1^, physical forces may influence the adsorption mechanism.

The dimensionless constant separation factor *R*_L_ is used to predict whether an adsorption system is favorable or unfavorable [32], and it can express the essential characteristics of the Langmuir isotherm. The formula for the separation factor *R*_L_ is given by Equation (13).

(13) $$R_{L}=\frac{1}{1+K_{L}C_{0}}$$

where *C*_0_ represents the initial concentration of U(VI) (mg·L^−1^), and *K*_L_ stands for the Langmuir adsorption equilibrium constant (L·mg^−1^).

### 2.10. Thermodynamic Studies

The adsorption thermodynamics parameters are analyzed by Equations (14) and (15) [9].

(14) $$lnK_{d}=\frac{\Delta S^{{}^{\circ}}}{R}-\frac{\Delta H^{{}^{\circ}}}{RT}$$

(15) $$\Delta G^{{}^{\circ}}=\Delta H^{{}^{\circ}}-T\Delta S^{{}^{\circ}}$$

where *K*_d_ is the distribution coefficient (mL∙g^−1^) of U(VI), *T* is the absolute temperature (K), and *R* is the ideal gas constant (8.314 J∙mol^−1^∙K^−1^).

### 2.11. Desorption and Regeneration Experiments

To assess the reusability of Fe_3_O_4_-CMC/G1.5 and Fe_3_O_4_-P-CMC/G1.5(1–3), consecutive adsorption–desorption cycles were conducted in Erlenmeyer flasks using 0.005 g of Fe_3_O_4_-P-CMC/G1.5-2 and 10 mg∙L^−1^ of U(VI) solution. The suspension was continuously stirred for 120 mins at a pH of 5.5 and a temperature of 298.15 K. After adsorption, the U(VI)-loaded Fe_3_O_4_-P-CMC/G1.5-2 was desorbed in 50 mL of HCl solutions, with concentrations ranging from 0.1 to 1.5 mol∙L^−1^, while being stirred at 180 rpm for 90 mins at 298.15 K. Subsequently, the suspension was centrifuged, and the U(VI)-loaded Fe_3_O_4_-P-CMC/G1.5-2 solid was washed with deionized water until neutrality was achieved. It was then freeze-dried in a freeze dryer at 207 K and reused for subsequent tests. The adsorption–desorption cycles were repeated five times, and the concentration of U(VI) in the aqueous phase was determined as previously described.

## 3. Results and Discussion

### 3.1. Characterization of Fe_3_O_4_-P-CMC/PAMAM

Figure S1 shows the ^1^H NMR spectra of G0.5 and G1.5 (see Supporting Information). The proton resonances of -COOCH_3_ at 3.67 ppm for both molecules belong to the terminal groups of the dendrimers. The presence of methylene peaks of -NCH_2_CH_2_CO at 2.73–2.79 ppm displayed a successful connection between ethylenediamine and methyl acrylate through the Michael addition reaction. The as-prepared G0.5 subsequently formed a NH_3_-terminal intermediate G1.0 by amidation, and the Michael addition reaction was repeated to give G1.5, as evidenced by the presence of -CONHCH_2_ at 3.30–3.27 ppm and -CH_2_CONH at 2.36–2.32 ppm. 

**Figure 1:**
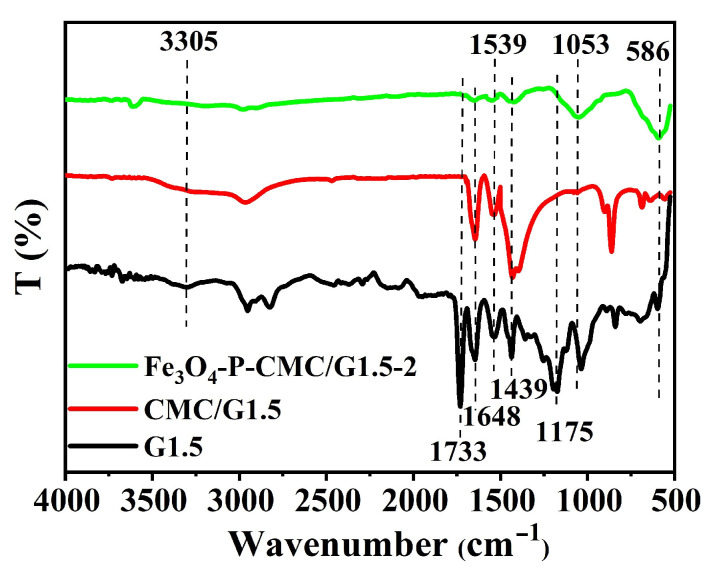
FT-IR spectra of G1.5, CMC/G1.5, and Fe_3_O_4_-P-CMC/G1.5-2.

Figure 1 shows the FT-IR spectra of G1.5, CMC/G1.5 and Fe_3_O_4_-P-CMC/G1.5-2 nanoparticles. In pure PAMAM, a peak at 3305 cm^−1^ is ascribed to the N-H stretching vibration of amide. The presence of two peaks at 1733 and 1648 cm^−1^ is ascribed to the C=O stretching vibration of the terminal ester and amide (-COOCH_3_, -CONH-), respectively. A peak at 1539 cm^−1^ is assigned to the –NH bending vibration of the amide (-CONH-). Moreover, a peak at 1439 cm^−1^ is attributed to the C-N stretching vibration of the tertiary amine (N-(CH_2_)_3_). A peak at 1195 cm^−1^ is assigned to the C-O stretching vibration of the ester (-COO-). FT-IR spectra analysis for PAMAM indicated that the amino and ester groups were successfully converted into tertiary amines by the Michael addition and amide groups, respectively. The peak at 1735 cm^−1^ of ester disappeared in CMC/G1.5 and Fe_3_O_4_-P-CMC/G1.5-2, implying that G1.5 with terminal ester groups was successfully grafted onto CMC chains to form a three-dimensional network by the divergent strategy. The presence of two peaks at 1053 and 586 cm^−1^ is attributed to P-O and Fe-O, respectively, suggesting that phosphorylation and magnetic function were achieved in the as-prepared adsorbents. 

**Figure 2:**
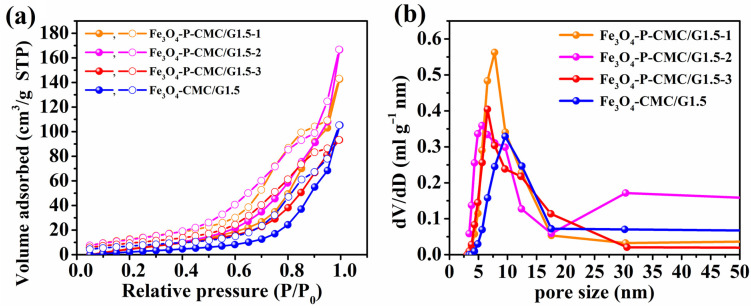
(**a**) N_2_ adsorption–desorption isotherms (solid sphere: adsorption; hollow dot: desorption) and (**b**) pore size distribution of Fe_3_O_4_-P-CMC/G1.5 series and Fe_3_O_4_-CMC/G1.5.

The adsorption–desorption isotherms of the as-prepared adsorbents under the N_2_ atmosphere are shown in Figure 2a. These adsorbents belong to typical IV isotherm curves, indicating that the adsorbents have a similar porosity and morphology [33,34]. The surface areas of the Fe_3_O_4_-P-CMC/G1.5 series are 44.27, 43.31, 42.98 m^2^/g, respectively, which are higher than the 41.74 m^2^/g of Fe_3_O_4_-CMC/G1.5 attributed to the high surface area of PAMAM [35]. The spherical framework of PAMAM forms a self-supporting structure within CMC molecules. The surface areas of Fe_3_O_4_-P-CMC/PAMAM series adsorbents did not decrease significantly with increasing concentration of PAMAM, suggesting that the introduction of PAMAM species only leads to a trivial steric hindrance. As shown in Figure 2b, the pore size of the Fe_3_O_4_-P-CMC/PAMAM series ranges from 5.60 to 9.60 nm, in which Fe_3_O_4_-P-CMC/G1.5-2 has the minimum pore size of 5.60 nm. As a result, the as-prepared Fe_3_O_4_-P-CMC/PAMAM adsorbents are nanoparticles with mesopores that probably exist on the external and internal surfaces of the materials. 

**Figure 3:**
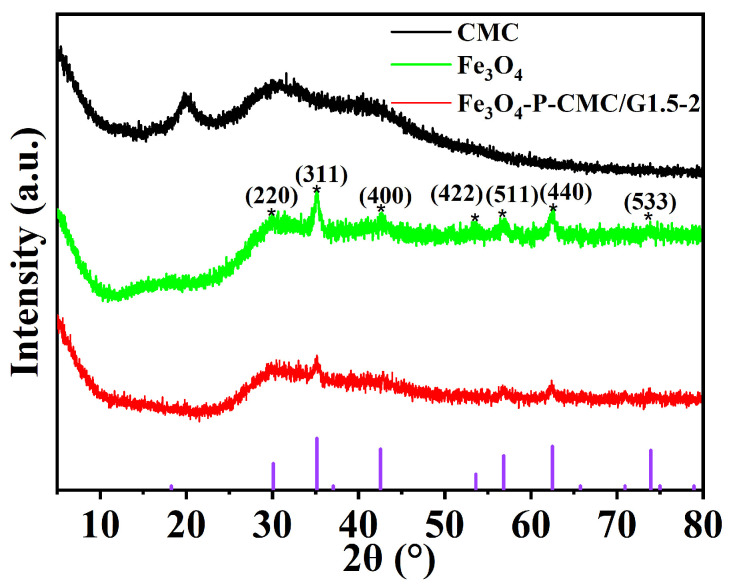
XRD patterns of CMC, Fe_3_O_4_, and Fe_3_O_4_-P-CMC/G1.5-2. (* represents the characteristic diffraction peaks of Fe_3_O_4_).

As shown in Figure 3, in terms of the XRD patterns of Fe_3_O_4_ and Fe_3_O_4_-P-CMC/G1.5-2, six characteristic diffraction peaks (311), (400), (422), (511), (440), and (533) can be observed for both, indicating that Fe_3_O_4_ was successfully inserted into CMC/G1.5 networks with consolidation by the intermolecular force [36]. The XRD pattern of the CMC exhibits a strong diffraction peak at 2θ = 20°, which completely disappears in the as-prepared Fe_3_O_4_-P-CMC/G1.5-2. As expected, PAMAM was well dispersed into the CMC molecules, leading to a homogeneous phase in which the crystalline orientation in the CMC was disordered by the penetration of PAMAM. 

**Figure 4:**
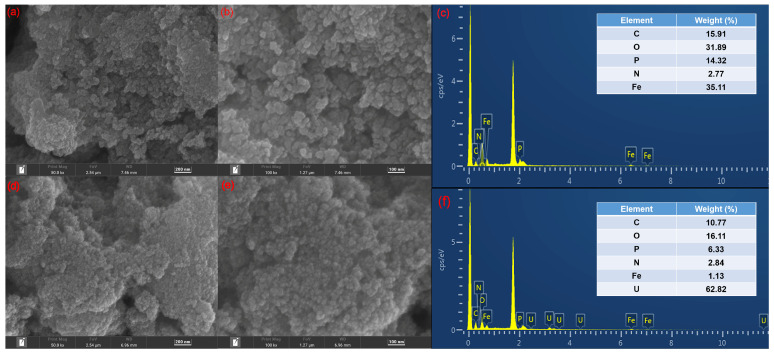
(**a**,**b**) SEM images and (**c**) EDS spectrum of Fe_3_O_4_-P-CMC/G1.5-2; (**d**,**e**) SEM images and (**f**) EDS spectrum of Fe_3_O_4_-P-CMC/G1.5-2-U.

The morphologies of Fe_3_O_4_-P-CMC/G1.5-2 nanoparticles before and after U(VI) adsorption were determined by SEM. As shown in Figure 4a,b, there are a myriad of nanoscale regular round flakes agglomerating and packing together with an average particle size ranging from 20 to 50 nm. The morphologies result in abundant exposed functional groups coupling with appreciable surface areas for Fe_3_O_4_-P-CMC/PAMAM series adsorbents, which ensure a high adsorption capacity for U(VI). Figure 4c depicts the EDS spectrum of Fe_3_O_4_-P-CMC/G1.5-2 before U(VI) adsorption. Strong peak signals of Fe, O, C, and P can be observed, indicating that Fe_3_O_4_ and -PO_3_ were successfully incorporated into Fe_3_O_4_-P-CMC/G1.5-2. Based on the EDS spectrum, the weight percentages of C, O, P, N, and Fe are 15.91%, 31.89%, 14.32%, 2.77%, and 35.11%, respectively. As shown in Figure 4d,e, the morphological structures of Fe_3_O_4_-P-CMC/G1.5-2-U are similar to those of the pristine before the adsorption of U(VI), whereas a relatively smaller particle size is observed. As shown in Figure 4f, from the EDS spectrum of Fe_3_O_4_-P-CMC/G1.5-2-U, the weight percentages of C, O, P, N, Fe, and U are 10.77%, 16.11%, 6.33%, 2.84%, 1.13%, and 62.82%, respectively. Fe_3_O_4_-P-CMC/G1.5-2-U exhibits the highest amount of U, but that of Fe decreased dramatically, suggesting that the functional groups of Fe_3_O_4_-P-CMC/G1.5-2 prefer conjugation with U(VI) to Fe_3_O_4_.

### 3.2. Effect of pH Values

**Figure 5:**
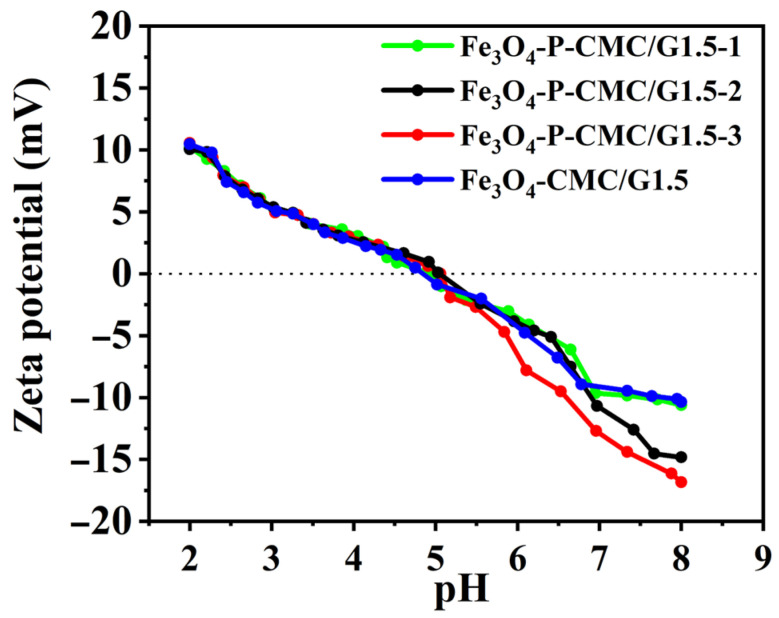
Zeta potential of Fe_3_O_4_-P-CMC/PAMAM.

The pH value of the solution significantly affected the U(VI) adsorption efficacy of the adsorbent. The species of uranyl ions in the solution and surface features of the adsorbent simultaneously determined the adsorption behavior of uranyl ions [37]. Figure 5 shows the surface charges of four adsorbents at pH 2–8. As the pH increases, the Zeta potential of Fe_3_O_4_-P-CMC/G1.5-1, Fe_3_O_4_-P-CMC/G1.5-2, Fe_3_O_4_-P-CMC/G1.5-3, and Fe_3_O_4_-CMC/G1.5 gradually decreases, and the pH zero-point charge (pH_ZPC_) value is 5.5. Under the condition of pH < pH_ZPC_, the adsorbent surface carries a positive charge, and U(VI) exists mainly in solution in the form of ${\mathrm{UO}}_{2}^{2+}$. The presence of electrostatic repulsion and competitive H^+^ in the aqueous solutions is not conducive to the enrichment of ${\mathrm{UO}}_{2}^{2+}$ on the adsorbent surface with a positive charge, resulting in a decrease in adsorption capacity. When pH > pH_ZPC_, as a result of the deprotonation effect on the surface of the adsorbent, it carries a certain amount of negative charge. 

**Figure 6:**
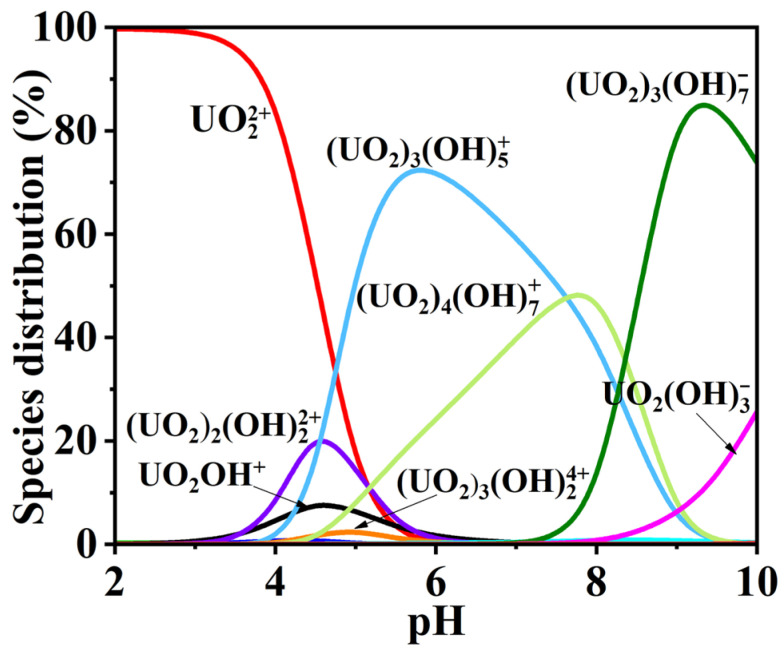
Distribution of U(VI) species at different pH values obtained by Visual MINEQL 3.0.

The distribution of U(VI) species in aqueous solution can be obtained from Visual MINEQL 3.0 based on the Nuclear Energy Agency (NEA) database [38]. As shown in Figure 6, the components containing uranium in the solution exist mainly in the forms of ${\mathrm{UO}}_{2}^{2+}$, (UO_2_)_2_(OH${)}_{2}^{2+}$, (UO_2_)_4_(OH)_7_)^+^ and (UO_2_)_3_(OH)_5_)^+^ [39]. Electrostatic attraction will promote the fixation of positively charged uranium (VI) on the negatively charged adsorbent surface [40].

**Figure 7:**
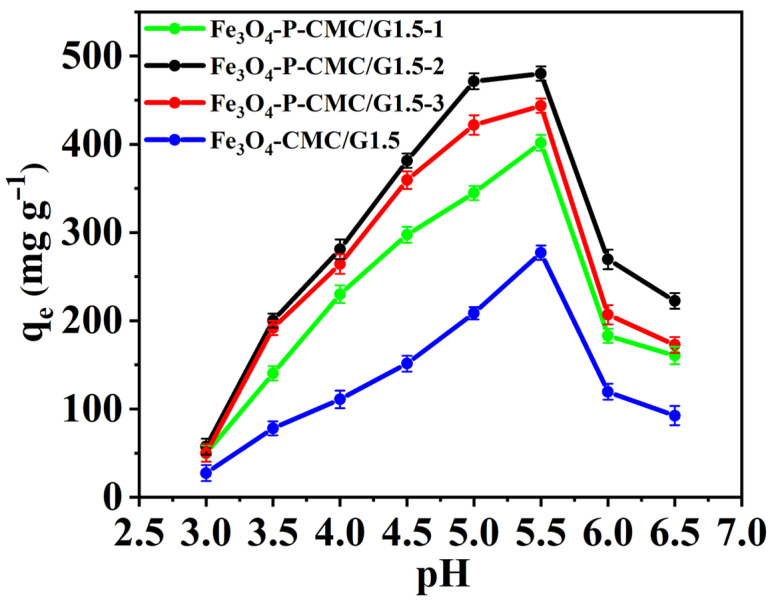
**The** effect of pH on the adsorption of Fe_3_O_4_-P-CMC/PAMAM (*C*_0_ = 50 mg·L^−1^, *T* = 298.15 K, *m* = 0.005 g, *V* = 50 mL, *t* = 170 min).

To study the influence of pH values on the adsorption performance of these materials, adsorption experiments were carried out in the range of pH 3.0–6.5 considering the fact that uranium ions usually precipitate at a higher pH [41,42]. Figure 7 shows the influence of pH values on the adsorption performance of Fe_3_O_4_-P-CMC/PAMAM. The amount of adsorbed uranyl ions in Fe_3_O_4_-P-CMC/G1.5-1, Fe_3_O_4_-P-CMC/G1.5-2, Fe_3_O_4_-P-CMC/G1.5-3, and Fe_3_O_4_-CMC/G1.5 increased consistently with increasing pH to 5.5 and was followed by a dramatic decrease. It was noted that the degree of protonation of functional groups on the adsorbent decreased, leading to a gradual enhancement of complexation and electrostatic ability and a significant increase in adsorption capacity. At pH 5.5, the as-prepared adsorbents exhibited a respective maximum adsorption capacity, in which that of Fe_3_O_4_-P-CMC/G1.5-1, Fe_3_O_4_-P-CMC/G1.5-2, Fe_3_O_4_-P-CMC/G1.5-3, and Fe_3_O_4_-CMC/G1.5 was 401.7 mg·g^−1^, 480.2 mg·g^−1^, 443.7 mg·g^−1^ and 277.2 mg·g^−1^, respectively. As the degree of grafting increases, the adsorption capacity of the adsorbent gradually increases and then decreases. Additionally, Fe_3_O_4_-P-CMC/G1.5-2 presents a maximum adsorption performance, suggesting that the optimal degree of PAMAM grafting in this study is G1.5-2.

### 3.3. Adsorption Kinetics

**Figure 8:**
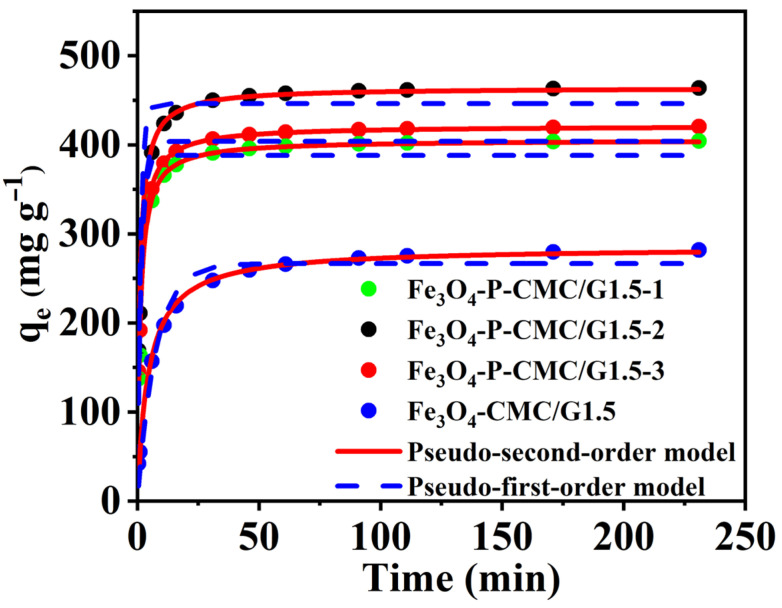
Adsorption kinetic curves of U(VI) on Fe_3_O_4_-P-CMC/PAMAM (*C_0_* = 50 mg·L^−1^, *T* = 298.15 K, *m* = 0.005 g, *V* = 50 mL, pH = 5.5).

Figure 8 shows the influence of contact time on the adsorption of U(VI) by Fe_3_O_4_-P-CMC/PAMAM. The adsorption capacity of Fe_3_O_4_-P-CMC/PAMAM increased sharply in 30 min, in which over 90% of the maximum adsorption capacity took place in this region, then slowly increased until a saturated adsorption state was reached in 2 h. It is possible that Fe_3_O_4_-P-CMC/PAMAM adsorbents are highly water permeable and the construction of mesopore three-dimensional scaffolds based on PAMAM dendrimers is well defined. In addition, three kinetic models, i.e., the pseudo-first-order model, pseudo-second-order model, and intraparticle diffusion model, were used to estimate the adsorption kinetic data to explain its adsorption mechanism. The three models are expressed using Equations (6)–(8) [43,44,45]. The fitting results are shown in Tables S1 and S2.

The results showed that the correlation coefficient *R*^2^ (0.96, 0.95, 0.95, and 0.93) of the pseudo-first-order model of Fe_3_O_4_-P-CMC/PAMAM was significantly lower than that of the pseudo-second-order model (0.99, 0.99, 0.99, and 0.99). In addition, the theoretical equilibrium adsorption capacity (*q*_e,cal_ = 284.89 mg·g^−1^, 405.40 mg·g^−1^, 464.00 mg·g^−1^, 421.27 mg·g^−1^) and the experimental equilibrium adsorption capacity (*q*_e,exp_ = 281.82 mg·g^−1^, 404.30 mg·g^−1^, 463.81 mg·g^−1^, 420.32 mg·g^−1^) of Fe_3_O_4_-P-CMC/G1.5 are closer, suggesting that the adsorption of U(VI) by Fe_3_O_4_-P-CMC/PAMAM was described more accurately by the pseudo-second-order kinetic model. More specifically, the U(VI) adsorption process on Fe_3_O_4_-P-CMC/PAMAM was controlled by a chemical process, suggesting that multiple mechanisms were probably involved in the adsorption of U(VI) on Fe_3_O_4_-P-CMC/PAMAM ascribed to oxygen-rich functional groups, such as -PO_3_H_2_ [46].

**Figure 9:**
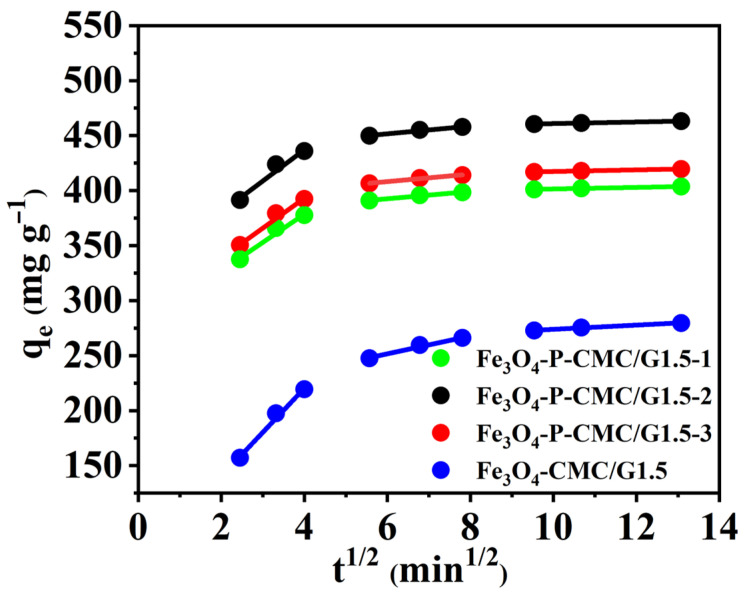
Intraparticle diffusion model plots of U(VI) adsorption on Fe_3_O_4_-P-CMC/PAMAM (*C*_0_ = 50 mg·L^−1^, *T* = 298.15 K, *m* = 0.005 g, *V* = 50 mL, pH = 5.5).

The intraparticle diffusion model of the Fe_3_O_4_-P-CMC/PAMAM adsorption of U(VI) was used to analyze the controlling steps of the adsorption rate. As shown in Figure 9, the relation of the fitting curve between *q*_t_ and *k*_int_ reveals that the adsorption process of U(VI) by Fe_3_O_4_-P-CMC/PAMAM can be depicted in three distinct regions. The diffusion rate of each follows the order of *k*_int,1_ > *k*_int,2_ > *k*_int,3_ which represents the rapid adsorption stage, slow adsorption stage, and equilibrium stage, respectively. In the initial rapid-uptake region, the reactive sites exposed on the exterior surface of Fe_3_O_4_-P-CMC/PAMAM captured U(VI) at a fast rate. After uranyl ions subsequently penetrated deeply into the pores, U(VI) was then adsorbed at a relatively slow rate by the reactive sites exposed on the interior surface. The limiting factor of rate depression is attributed to intraparticle diffusion in this stage [47]. Finally, the active sites of Fe_3_O_4_-P-CMC/PAMAM became fully occupied, leading to a near-zero diffusion rate constant, and signifying that the adsorption had attained equilibrium. As a result, the U(VI) adsorption by Fe_3_O_4_-P-CMC/PAMAM is predominantly attributed to robust surface interactions or chemisorptive bonding. 

### 3.4. Adsorption Isotherms

**Figure 10:**
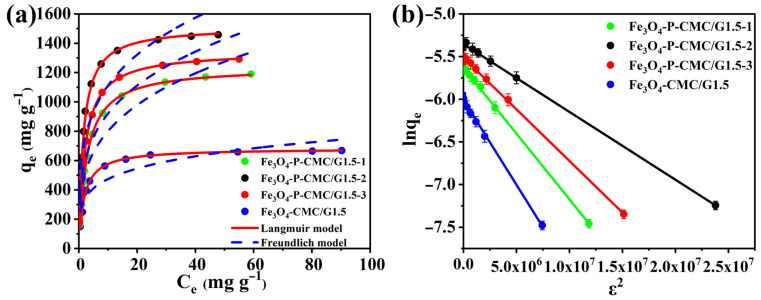
Adsorption isotherm curves of U(VI) on Fe_3_O_4_-P-CMC/PAMAM. (**a**) Langmuir and Freundlich models; (**b**) D-R isotherm model (*m* = 0.005 g, *V* = 50 mL, pH = 5.5, *T* = 298.15 K, *t* = 170 min).

Figure 10a,b display the U(VI) adsorption isotherms of Fe_3_O_4_-P-CMC/PAMAM. At pH 5.5 and 298.15 K, the ability to adsorb uranyl ions heightened as the initial concentration of U(VI) in the solution was raised from 10 mg·L^−1^ to 200 mg·L^−1^. The adsorption behavior was investigated by the Langmuir model, Friedrich model, and Dubinin–Radushkevich (D-R) model. The three models are expressed using Equations (9)–(11), respectively. Dynamic simulations were performed on the basis of experimental data for corresponding isotherm models to obtain the slopes and intercepts from curve fittings. The values of the calculated parameters are tabulated in Tables S3 and S4 [48].

Compared to the Friedrich model, the absorption of U(VI) in Fe_3_O_4_-P-CMC/G1.5 series is better delineated by the Langmuir isotherm model, which has higher correlation coefficients (*R*^2^ > 0.99), indicating a monolayer adsorption process [49]. Moreover, the maximum Langmuir adsorption capacity of Fe_3_O_4_-P-CMC/G1.5 series for U(VI) was 1238.49 mg·g^−1^ (G1.5-1), 1513.47 mg·g^−1^ (G1.5-2), and 1344.14 mg·g^−1^ (G1.5-3), whereas that of Fe_3_O_4_-CMC/G1.5 was 681.31 mg·g^−1^. The results indicate that Fe_3_O_4_-P-CMC/G1.5-2 has the highest affinity for U(VI). On the other hand, the adsorption isotherms of U(VI) onto Fe_3_O_4_-P-CMC/G1.5 series were simulated by the D-R model where the correlation coefficients (*R*^2^) were found to be greater than 0.99. *E*_DR_ values ranging from 12.34 to 15.62 kJ·mol^−1^ were between 8 and 16 kJ·mol^−1^, suggesting chemisorption [50].

### 3.5. Adsorption Thermodynamic

**Figure 11:**
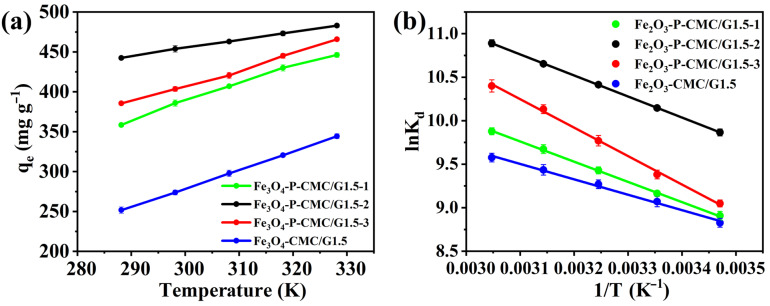
(**a**) Effect of Fe_3_O_4_-P-CMC/PAMAM temperature on adsorption of U(VI). (**b**) Curve fitting of ln*K*_d_ vs. 1/*T* (*C*_0_ = 50 mg·L^−1^, *m* = 0.005 g, *V* = 50 mL, pH = 5.5, *t* = 170 min).

The influence of temperature on U(VI) adsorption by Fe_3_O_4_-P-CMC/PAMAM was studied in the temperature range from 288.15 to 328.15 K (Figure 11a). As temperature increases, the adsorption capacity of Fe_3_O_4_-P-CMC/PAMAM for U(VI) steadily increases, indicating that the adsorption process is a critically temperature-dependent behavior and ostensibly endothermic. It is indicative of the fact that elevated temperatures of the aqueous solution induce collisions between the surface of the adsorbents and uranyl ions, increase kinetic energy, and therefore improve the diffuse coefficient. 

Gaining deeper insights into the thermodynamic properties of the Fe_3_O_4_-P-CMC/PAMAM adsorption process is crucial. The thermodynamic parameters of the adsorption process, such as standard entropy (Δ*S*°), enthalpy (Δ*H*°), and Gibbs free energy (Δ*G*°) were calculated from the intercept and slope by fitting linear ln*K*_d_ vs. 1/*T* (Figure 11b). By using Equations (14) and (15), the values of Δ*G*° are obtained as listed in Table S6. As shown in Table S6, the positive values of Δ*H*° indicate that the adsorption process of Fe_3_O_4_-P-CMC/PAMAM towards U(VI) is endothermic [51,52]. The positive values of Δ*S*° show that the occurrence of U(VI) uptake within the solid solution interface increases the degree of freedom of the ions and the disorder of the system, leading to an increase in entropy. 

In addition, the negative values of Δ*G*° indicate that the adsorption process of Fe_3_O_4_-P-CMC/PAMAM for U(VI) is spontaneous. As can be noted, while the increase in temperature from 288.15 K to 328.15 K is accompanied by a concomitant decrease in Δ*G*° ranging from *ca*. −21~−23 kJ·mol^−1^ to *ca*. −26~−29 kJ·mol^−1^, the adsorption process takes place more easily at higher temperatures. 

### 3.6. Effect of Competitive Ions

**Figure 12:**
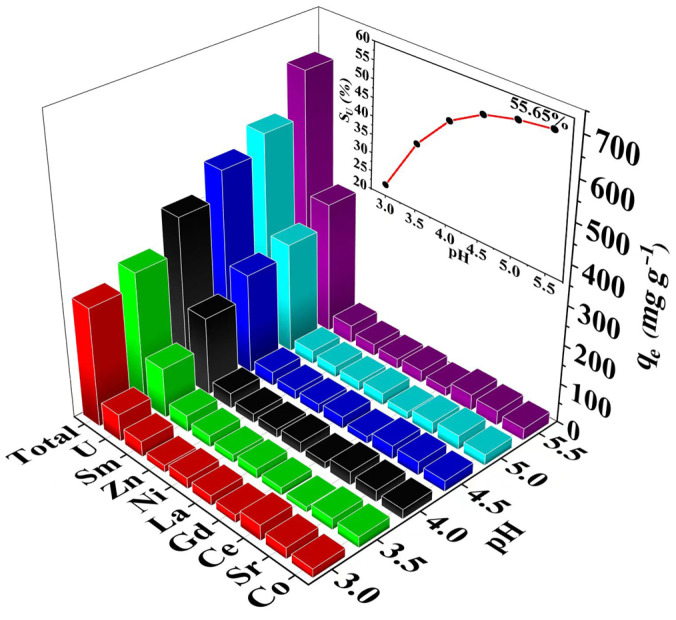
pH effect on the selective adsorption of Fe3O4-P-CMC/G1.5-2 towards U(VI) accompanied by competing cations (*C*_0_ = 50 mg·L^−1^, *m* = 0.005 g, *V* = 50 mL, *T* = 298.15 K).

The efficient and selective adsorption of uranium from nuclear wastewater has always been a major challenge in practical applications. The U(VI)-selective adsorption of Fe_3_O_4_-P-CMC/G1.5-2 was investigated with eight coexisting cations, including Zn^2+^, Ni^2+^, Co^2+^, Sr^2+^, Ce^3+^, Gd^3+^, La^3+^, and Sm^3+^. As shown in Figure 12, *q*_e, total_ and *q*_e,U_ progressively rise as the pH value elevates from 3.0 to 5.5, and the adsorption capacities for uranium reach maximum values of 650 and 340 mg·g^−1^ at pH 5.5, respectively. The approximately 55.6% adsorption capability of Fe_3_O_4_-P-CMC/G1.5-2 for U(VI) indicates that it exhibits a relatively higher selectivity for U(VI) among multi-ion solutions.

**Figure 13:**
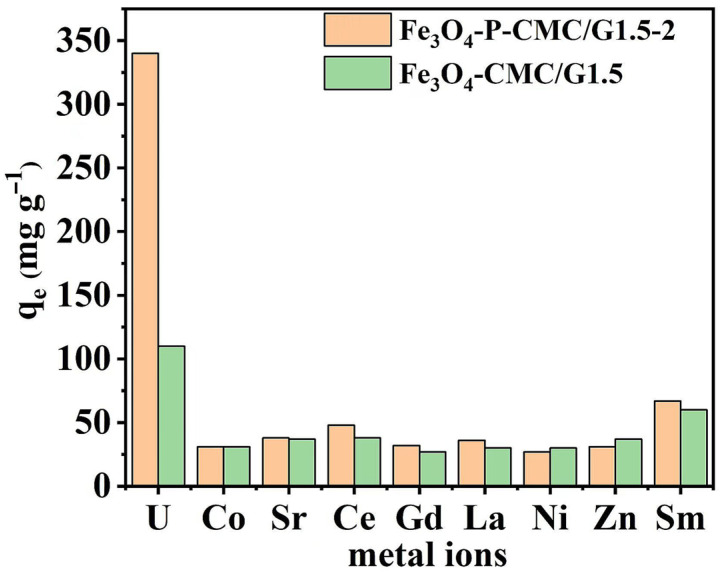
Distinct adsorption comparison of coexistent cations by Fe_3_O_4_-CMC/G1.5 and Fe_3_O_4_-P-CMC/G1.5-2 (*C*_0_ = 50 mg·L^−1^, *m* = 0.005 g, *V* = 50 mL, *T* = 298.15 K, pH = 5.5).

In addition, Figure 13 shows a comparison between Fe_3_O_4_-CMC/G1.5 and Fe_3_O_4_-P-CMC/G1.5-2 in terms of the selective performance of a multi-ion solution at pH 5.5. Compared to Fe_3_O_4_-CMC/G1.5, the selectivity coefficients (*S*_U(VI)/M(x)_) of Fe_3_O_4_-P-CMC/G1.5-2 are dramatically higher among competing cations, especially in terms of Ce (III), Gd (III), La (III), and Sm (III) (Table S7). As a result, Fe_3_O_4_-P-CMC/G1.5-2 is considered to be a more viable contender for the selective adsorption of U(VI) within a multi-ion solution. 

### 3.7. Desorption and Reusability

**Figure 14:**
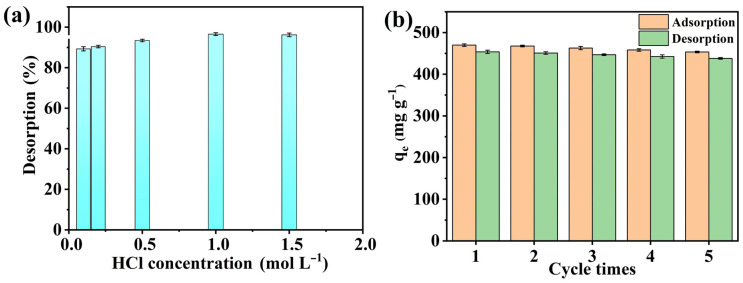
(**a**) Desorption efficiency of different HCl concentrations. (**b**) Regenerations of Fe_3_O_4_-P-CMC/G1.5-2 5 times.

Taking into account economic and ecological factors for an eco-friendly adsorbent, the investigation of regeneration performance is of great significance. After adsorption, desorption eluent tests of Fe_3_O_4_-P-CMC/G1.5-2 were first carried out with 1 M HCl, 1M H_2_SO_4_, 1 M HNO_3_, 1 M Na_2_CO_3_, and 0.1 M EDTA-Na_2_, respectively. The desorption efficacy of HCl was found in a range from 92% to 96%, which was the highest among the five solutions, suggesting that HCl is the best choice for the desorption of U(VI) from Fe_3_O_4_-P-CMC/G1.5-2 (Figure S2 in Supporting Information). Figure 14a shows the desorption efficiency under a concentration of HCl ranging from 0.1 M to 1.5 M. The desorption rate of Fe_3_O_4_-P-CMC/G1.5-2 is positively related to the HCl concentration, indicating that acid treatment is applicable to the desorption of U(VI) for Fe_3_O_4_-P-CMC/G1.5-2. The results indicate that a 1 M HCl solution with the highest desorption efficiency should be selected for U(VI) desorption [53,54].

As shown in Figure 14b, more than 90% of U(VI) could be easily desorbed from Fe_3_O_4_-P-CMC/G1.5-2, which retained a high adsorption capability after undergoing five adsorption–desorption cycles. The chemical tenability of Fe_3_O_4_-P-CMC/PAMAM in desorption circumstances virtually guarantees that the high-reusability performance is reliable and available. 

### 3.8. Possible Adsorption Mechanism

**Figure 15:**
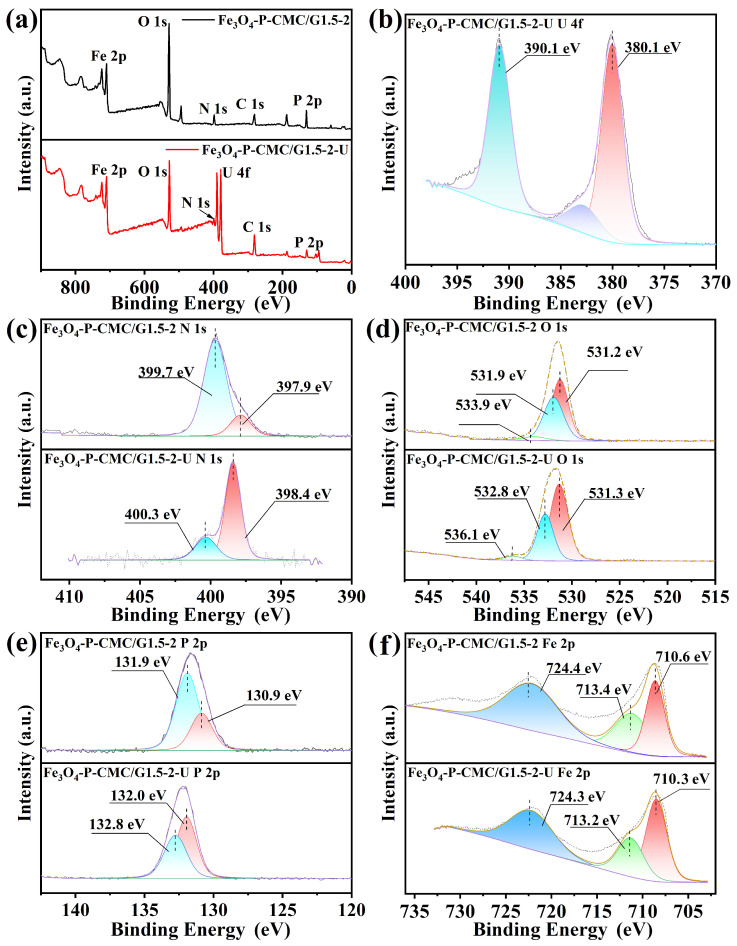
(**a**) XPS survey spectra of Fe_3_O_4_-P-CMC/G1.5-2 and Fe_3_O_4_-P-CMC/G1.5-2-U; (**b**) U 4f XPS spectrum of Fe_3_O_4_-P-CMC/G1.5-2-U; (**c**) N 1s XPS spectra of Fe_3_O_4_-P-CMC/G1.5-2 and Fe_3_O_4_-P-CMC/G1.5-2-U; (**d**) O 1s XPS spectra of Fe_3_O_4_-P-CMC/G1.5-2 and Fe_3_O_4_-P-CMC/G1.5-2-U; (**e**) P 2p XPS spectra of Fe_3_O_4_-P-CMC/G1.5-2 and Fe_3_O_4_-P-CMC/G1.5-2-U; (**f**) Fe 2p XPS spectra of Fe_3_O_4_-P-CMC/G1.5-2 and Fe_3_O_4_-P-CMC/G1.5-2-U. (Different colors of these splitted peaks represent distinct bonding types.).

The mechanism of adsorption of U(VI) on Fe_3_O_4_-P-CMC/G1.5-2 was elucidated by XPS. As shown in Figure 15a, the prominent appearance of U 4f accounts for the presence of U(VI) on Fe_3_O_4_-P-CMC/G1.5-2. Figure 15b depicts the high-resolution spectrum of U 4f, where bimodal peaks at 380.1 eV and 390.1 eV belong to U 4f_7/2_ and U 4f_5/2_, respectively, suggesting two reaction sites within Fe_3_O_4_-P-CMC/G1.5-2 [55]. As depicted in Figure 15c, the high-resolution N 1s spectrum of Fe_3_O_4_-P-CMC/G1.5-2 was divided into two peaks with binding energies of 399.7 eV and 397.9 eV, which correspond to NH_2_ and NH-C=O, respectively. On the other hand, the high-resolution N 1s spectrum of Fe_3_O_4_-P-CMC/G1.5-2-U was separated into two peaks at 400.3 eV and 398.4 eV, both of which shifted to a region of higher bonding energy, suggesting that nitrogen species were involved in complexation with uranyl ions. The high-resolution O 1s spectra of Fe_3_O_4_-P-CMC/G1.5-2 resolved into three peaks ascribed to C-O-P at 533.9 eV, P-OH at 532.3 eV, and P=O at 531.2 eV (Figure 15d). Both the binding energy values and areas of these O donor functional groups for Fe_3_O_4_-P-CMC/G1.5-2-U reveal perceived variations after U(VI) uptake. The high-resolution P 2p spectra of these adsorbents were divided into two peaks (Figure 15e). The P 2p peaks of Fe_3_O_4_-P-CMC/G1.5 consisted of P 2p_1/2_ at 131.9 eV and P 2p_3/2_ at 130.9 eV that individually shifted to 132.8 eV and 132.0 eV with changes in relative proportions. Thus, this difference implies that phosphate groups are strongly involved in the adsorption of U(VI) [56,57]. Figure 15f displays the high-resolution spectrum of Fe 2p for Fe_3_O_4_-P-CMC/G1.5-2, in which bimodal peaks with binding energies at 710.8 eV and 724.2 eV belong to Fe 2p_3/2_ and Fe 2p_1/2_, respectively. Furthermore, the Fe 2p_3/2_ peak could be split into 710.6 eV and 713.4 eV, which corresponded to the bonding energies of Fe (III) and Fe (II), respectively. After adsorption, the bonding energies of Fe 2p_1/2_ and Fe 2p_3/2_ did not change dramatically, indicating that Fe_3_O_4_ did not participate in the conjugation with uranium species during the adsorption process. Furthermore, the high-resolution C 1s spectra display three peaks, 286.3 eV for the O-C-O and N-C=O bonds, 284.5 eV for the C-O-P and C-OH bonds, and 282.8 eV for the -CH_2_ and -C-NH_2_ bonds (Figure S3). Different degrees of the positions and intensities of these peaks vary dramatically after the adsorption of U(VI). In light of the preceding analysis, the phosphonate and amino sites mounted on the surface of Fe_3_O_4_-P-CMC/G1.5 contributed significantly to the complexation with U(VI). 

## 4. Conclusions

In this work, a series of magnetic nanoparticle adsorbents were successfully prepared by crosslinking the PAMAM dendrimer and carboxymethyl chitosan, followed by phosphorylating the hydroxyl and amido groups by loading them with magnetic ferric oxide nanoparticles. The adsorbent demonstrated excellent performance in adsorbing U(VI), with a maximum adsorption capacity of 1513.47 mg·g^−1^ for Fe_3_O_4_-P-CMC/G1.5-2 at pH 5.5 and 298.15 K, indicating the excellent hydrophilic characteristics and spherical framework of the phosphorylated PAMAM dendrimer, which exhibited a high affinity and selectivity towards U(VI). The adsorption isotherm data closely aligned with the Langmuir model, suggesting that U(VI) uptake by Fe_3_O_4_-P-CMC/PAMAM occurred as a monolayer adsorption process. Similarly, the kinetic results were accurately represented by the pseudo-second-order kinetic model (*R*^2^ = 0.99, *q*_e,exp_ = 463.81 mg·g^−1^, *k*_2_ = 2.15 × 10^−2^ g·mg^−1^·min^−1^), further supporting the notion of a chemical adsorption mechanism for U(VI) adsorption on Fe_3_O_4_-P-CMC/PAMAM. The thermodynamic evaluation revealed that the adsorption process occurred spontaneously and was endothermic (ΔH° = 14.71 kJ·mol^−1^, ΔG° = −50.63 kJ·mol^−1^, 298. 15 K). The Fe_3_O_4_-P-CMC/PAMAM adsorbent demonstrated superior reusability, maintaining consistent adsorption efficiency and structural integrity even after five cycles. XPS analysis underscored the significance of phosphonate and amino groups in their interaction with uranyl ions, and confirmed the presence of dual peaks in the U 4f spectral region, with bonding energies at 380.1 eV and 390.1 eV corresponding to U 4f_7/2_ and U 4f_5/2_, respectively. This high level of performance, coupled with its stability and effective regeneration capabilities, suggests potential cost savings for Fe_3_O_4_-P-CMC/PAMAM in the context of wastewater remediation. It is plausible to regard Fe_3_O_4_-P-CMC/PAMAM as a potential option pertaining to the removal and recovery of U(VI) from radioactive waste streams. The effects of higher-generation PAMAM dendrimers on the adsorption performance of Fe_3_O_4_-P-CMC/PAMAM will be of great interest. Due to the nearly perfect hyperbranched topology structure of PAMAM dendrimers, the porous structure of a series of higher-generation PAMAM dendrimers would be strongly expected to impart more surface areas and reactive sites. Consequently, future work will be devoted to the systematical study of the effect of dendrimer generations on the adsorption performance of actinide elements.

## Data Availability

All data included in this study are available upon request by contacting the corresponding author.

## Supplementary Materials

The following supporting information can be downloaded at: https://www.mdpi.com/article/10.3390/nano14090810/s1. Figure S1: ^1^H NMR spectra of G0.5 and G1.5; Figure S2: Elution effect of different eluents on Fe_3_O_4_-CMC/G1.5 and Fe_3_O_4_-P-CMC/G1.5(1–3). (*m* = 5 mg, pH = 5.5, *C*_0_ = 50 mg·L^−1^, *t* = 170 min, *V* = 50 mL, *T* = 298.15 K); Figure S3: C 1s XPS spectra of Fe_3_O_4_-P-CMC/G1.5-2 and Fe_3_O_4_-P-CMC/G1.5-2-U; Table S1: Kinetic parameters for adsorption of U(VI) on Fe_3_O_4_-CMC/G1.5 and Fe_3_O_4_-P-CMC/G1.5(1–3) at different ratios; Table S2: Intra-particle diffusion parameters for adsorption of U(VI) on Fe_3_O_4_-CMC/G1.5 and Fe_3_O_4_-P-CMC/G1.5(1–3) at different ratios; Table S3: Isotherms parameters for adsorption of U(VI) on Fe_3_O_4_-CMC/G1.5 and Fe_3_O_4_-P-CMC/G1.5(1–3) at different ratios; Table S4: D-R parameters for adsorption of U(VI) on Fe_3_O_4_-CMC/G1.5 and Fe_3_O_4_-P-CMC/G1.5(1–3) at different ratios; Table S5: Langmuir separation factor *R_L_*; Table S6: The thermodynamic parameters for adsorption of U(VI) onto Fe_3_O_4_-CMC/G1.5 and Fe_3_O_4_-P-CMC/G1.5(1–3); Table S7: Distribution coefficient and selectivity coefficients of Fe_3_O_4_-CMC/G1.5 and Fe_3_O_4_-P-CMC/G1.5-2.

## Abbreviations

CMC		carboxymethyl chitosan
PAMAM		poly(amidoamine)
**Nomenclature**		
*C*	the constant proportional to the extent of the boundary layer thickness (mg·g^−1^)	
*C*_0_	the initial concentrations of the metal ion (mg∙L^−1^)	
*C*_e_	the equilibrium concentrations of the metal ion (mg∙L^−1^),	
*E*_DR_	the mean free energy of adsorption	
*k*_1_	the rate constants of pseudo-first-order model (min^−1^),	
*k*_2_	the rate constants of pseudo-second-order model (g∙mg^−1^∙min^−1^)	
*k*_int_	the rate constants of intraparticle diffusion kinetics model (mg·g^−1^·min^−0.5^)	
*K*_d_	the distribution coefficient (mL∙g^−1^)	
$K_{d}^{U(\mathrm{VI})}$	the distribution coefficients of uranyl ion	
$K_{d}^{M(x)}$	the distribution coefficients of other ions	
*K*_F_	Freundlich constants, indicating adsorption intensity	
*K*_L_	the Langmuir adsorption equilibrium constant (L·mg^−1^)	
*m*	the amount of the sorbent (g)	
*n*	Freundlich constants, indicating adsorption capacity	
*q*_e_	the amount of adsorption at equilibrium (mg∙g^−1^)	
*q*_e(U)_	the adsorption capacity of uranium (mg·g^−1^)	
*q*_e(Total)_	the total amount of adsorption for all multi-ions (mg∙g^−1^).	
*q*_DR_	the theoretical adsorption capacity (mol·g^−1^)	
*q*_m_	the maximum or saturated amount of adsorption (mg∙g^−1^)	
*q*_t_	U(VI) adsorption capacity at t (mg·g^−1^)	
R	the ideal gas constant (8.314 kJ·mol^−1^ K^−1^)	
*R*_L_	the dimensionless constant separation factor	
*S*_U_	degree of selectivity of the adsorbent towards uranium	
*S*_U(VI)/M(x)_	the selectivity coefficient of uranyl ions relative to competing ions	
*S*_r_	the relative selectivity coefficient	
*t*	the adsorption time (min)	
*T*	the absolute temperature (K).	
*V*	the volume of the solution (L)	
**Greek letters**		
*β*	the constant associated with the adsorption energy (mol^2^·kJ^−2^)	
*ε*	the Polanyi potential	
Δ*S*°	standard entropy (J·mol^−1^·K^−1^)	
Δ*H*°	standard enthalpy (kJ·mol^−1^)	
Δ*G*°	standard Gibbs free energy (kJ·mol^−1^)	
