# Supplementary material for: A Phosphorylated Dendrimer-Supported Biomass-Derived Magnetic Nanoparticle Adsorbent for Efficient Uranium Removal
Source: Nanomaterials (Basel). 2024 May 6;14(9):810. doi: 10.3390/nano14090810 (PMC11085421; doi:10.3390/nano14090810)
Supplement: Supplementary file 1 [file nanomaterials-14-00810-s001.zip › nanomaterials-2971723-supplementary.pdf]

Supporting Information on

A Phosphorylated Dendrimer-Supported Biomass-Derived Magnetic Nanoparticle Adsorbent for Efficient Uranium Removal

Mingyang Ma *, Qunyin Luo, Ruidong Han, Hongyi Wang, Junjie Yang and Chunyuan Liu *

State Key Laboratory of Nuclear Resources and Environment, East China University of Technology, Nanchang 330013, China

* Correspondence: m_ma@ecut.edu.cn (M.M.); 201861004@ecut.edu.cn (C.L.)

There are 8 pages with 3 Figures and 7 Tables in Supporting Information.

S1 Characterizations

Nuclear Magnetic Resonance (NMR) spectral data were acquired using a Varian-Mercury NMR instrument operating at a frequency of 300 MHz. Chemical shifts were referenced to the internal standards of chloroform ($CDCl_3$, with a hydrogen peak at δ = 7.26 ppm). The surface topography of the materials was examined using a field-emission scanning electron microscope (SEM, TESCAN MIRA LMS, TESCAN, Czech Republic). Fourier transform infrared spectra (FT-IR) were captured with a Nicolet iS5 FT-IR spectrometer (USA). The measurements of the specific surface area, average pore diameter, and pore volume for Fe_3O_4-P-CMC/PAMAM were conducted through N_2 adsorption and desorption tests at a temperature of 77 K. The specific surface area was determined by applying the classic Brunauer–Emmett–Teller (BET) theory. The pore size distribution was derived from the desorption arm of the isotherm using the Barrett–Joyner–Halenda (BJH) technique, with the aid of Quantachrome Auto sorb software provided by Quantachrome Instruments, located in Boynton Beach, Florida, USA. The crystalline structures were characterized using an X-ray diffractometer (Bruker D8 Advance) equipped with Cu Kα radiation, which scanned the specimens over an angular range of 20 to 80 degrees at a rate of 2°·min^{-1}. The surface elemental chemistry was analyzed using X-ray photoelectron spectroscopy (XPS, K-Alpha, Thermo Fisher Scientific, USA). Zeta potential measurements were conducted with a Particle Metrix flowing current potential analyzer (Stabino, Germany).

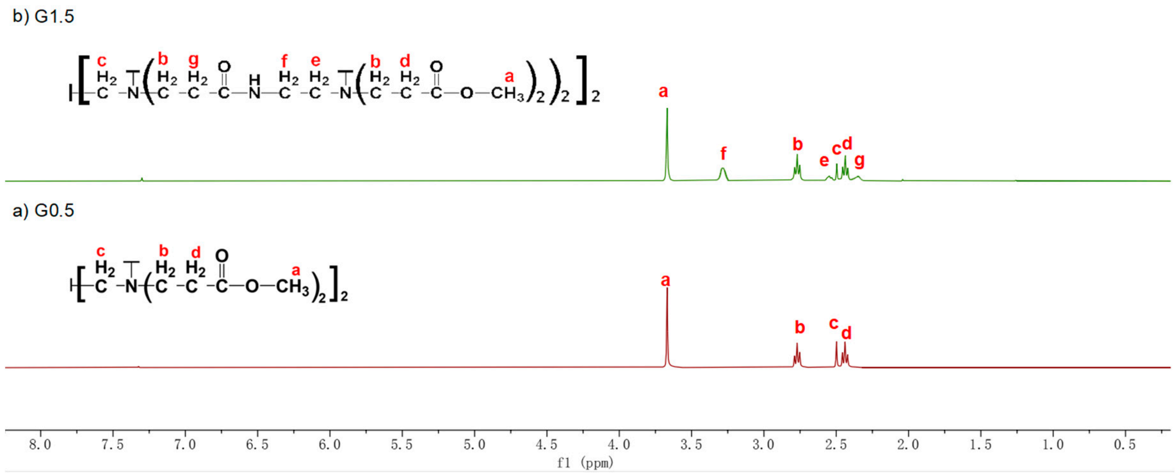

Figure S1. ^{1}H NMR spectra of G0.5 and G1.5.

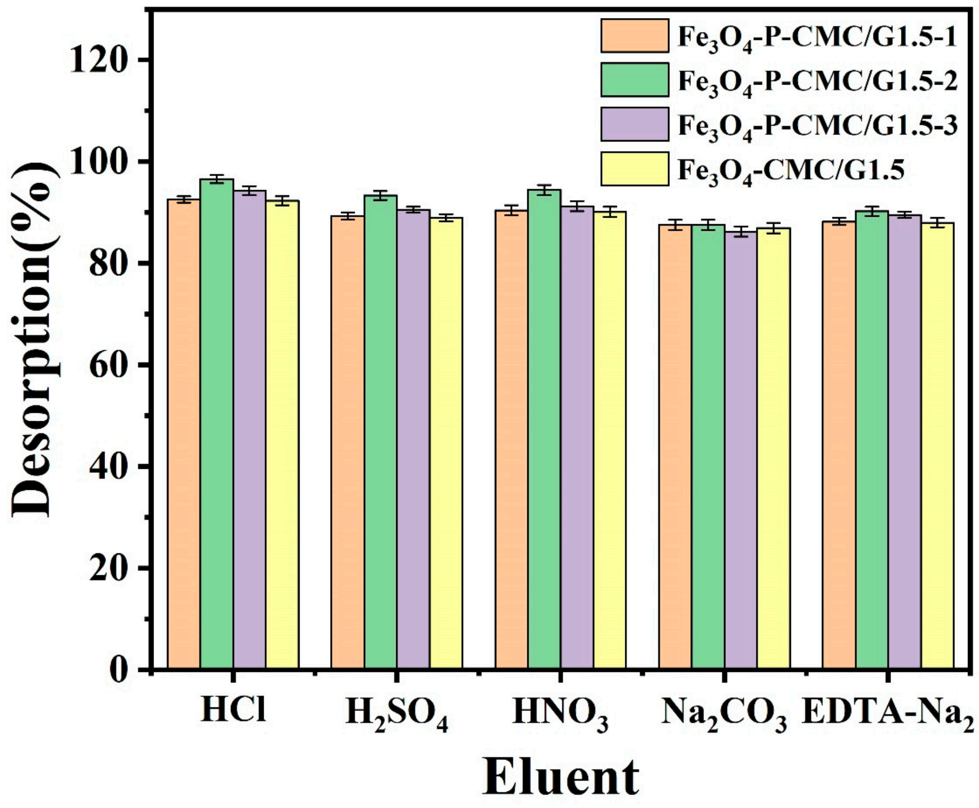

Figure S2. Elution effect of different eluents on Fe_3O_4-CMC/G1.5 and Fe_3O_4-P-CMC/G1.5 (1-3). (m=5 mg, pH=5.5, C_0=50 mg·L^{-1}, t=170 min, V=50 mL, T=298.15 K)

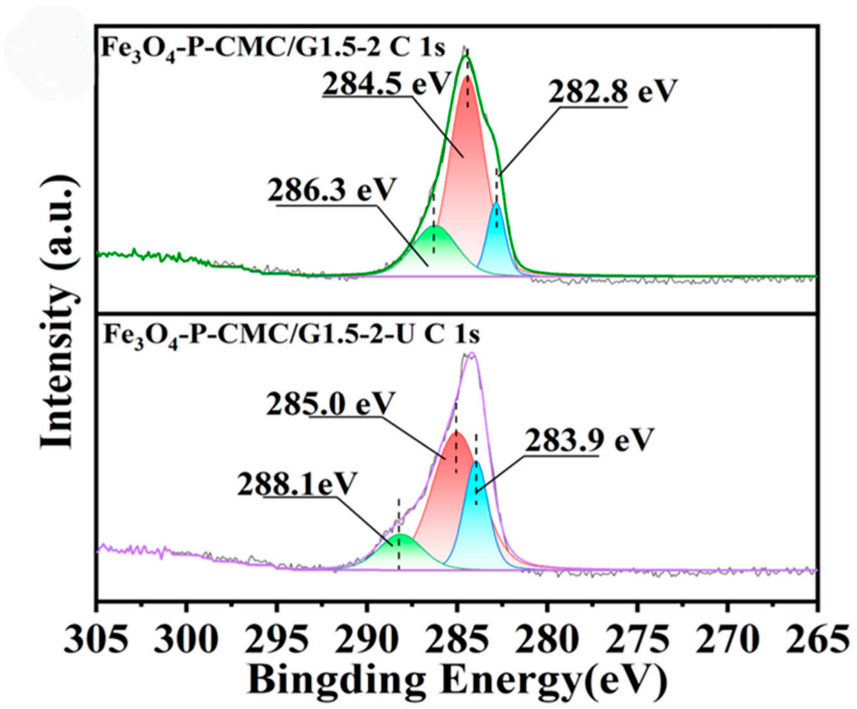

Figure S3. C 1s XPS spectra of Fe_3O_4-P-CMC/G1.5-2 and Fe_3O_4-P-CMC/G1.5-2-U.

Table S1. Kinetic parameters for adsorption of U(VI) on Fe_3O_4-CMC/G1.5 and Fe_3O_4-P-CMC/G1.5(1-3) at different ratios.

Sorbents	$q_{e,exp}$ (mg·g^{-1})	Pseudo-first-order kinetic			Pseudo-second-order kinetic		
		$q_{1,cal}$ (mg·g^{-1})	k_1 (min^{-1})	R^2	$q_{2,cal}$ (mg·g^{-1})	k_2 (g·mg^{-1}·min^{-1})	R^2
Fe_3O_4-CMC/G1.5	281.82	284.89	0.13	0.96	284.89	7.77×10^{-4}	0.99
Fe_3O_4-P-CMC/G1.5-1	404.30	405.40	0.67	0.95	405.40	2.10×10^{-2}	0.99
Fe_3O_4-P-CMC/G1.5-2	463.81	464.00	0.77	0.95	464.00	2.15×10^{-2}	0.99
Fe_3O_4-P-CMC/G1.5-3	420.32	421.27	0.64	0.93	421.27	2.11×10^{-2}	0.99

Table S2. Intra-particle diffusion parameters for adsorption of U(VI) on Fe_3O_4-CMC/G1.5 and Fe_3O_4-P-CMC/G1.5(1-3) at different ratios.

Sorbents	Intraparticle diffusion					
	k_{int}^1	R^2	k_{int}^2	R^2	k_{int}^3	R^2
Fe_3O_4-CMC/G1.5	39.38	0.99	8.11	0.99	1.91	0. 99
Fe_3O_4-P-CMC/G1.5-1	25.68	0.98	3.31	0.99	0.70	0.99
Fe_3O_4-P-CMC/G1.5-2	28.33	0.99	3.50	0.99	0.72	0.99
Fe_3O_4-P-CMC/G1.5-3	27.19	0.99	3.42	0.99	0.74	0.99

Table S3. Isotherms parameters for adsorption of U(VI) on Fe_3O_4-CMC/G1.5 and Fe_3O_4-P-CMC/G1.5(1-3) at different ratios.

Sorbents	Langmuir isotherm			Freundlich isotherm		
	q_m ($mg \cdot g^{-1}$)	K_L ($L \cdot mg^{-1}$)	R^2	K_F ($mol^{1-n} \cdot L^n \cdot g^{-1}$)	n	R^2
Fe_3O_4-CMC/G1.5	681.31	0.55	0.99	295.82	4.89	0.84
Fe_3O_4-P-CMC/G1.5-1	1238.49	0.37	0.99	411.87	3.46	0.89
Fe_3O_4-P-CMC/G1.5-2	1513.15	0.64	0.99	622.25	3.98	0.85
Fe_3O_4-P-CMC/G1.5-3	1344.14	0.47	0.99	490.25	3.66	0.88

Table S4. D-R parameters for adsorption of U(VI) on Fe_3O_4-CMC/G1.5 and Fe_3O_4-P-CMC/G1.5(1-3) at different ratios.

Sorbents	D-R isotherm		
	q_{DR}	$E_{D\text{-}R}$	R^2
Fe_3O_4-CMC/G1.5	0.0024	12.34	0.99
Fe_3O_4-P-CMC/G1.5-1	0.0036	14.29	0.99
Fe_3O_4-P-CMC/G1.5-2	0.0047	15.36	0.99
Fe_3O_4-P-CMC/G1.5-3	0.0040	13.61	0.99

Table S5. Langmuir separation factor R_L

C_0 (mg·L^{-1})	10	30	50	70	90	110	130	150	170	190	210
Fe_3O_4-CMC/G1.5	0.155	0.058	0.035	0.026	0.020	0.016	0.014	0.012	0.011	0.010	0.009
Fe_3O_4-P-CMC/G1.5-1	0.215	0.083	0.052	0.038	0.029	0.024	0.021	0.018	0.016	0.014	0.013
Fe_3O_4-P-CMC/G1.5-2	0.135	0.050	0.030	0.022	0.017	0.014	0.011	0.010	0.009	0.008	0.007
Fe_3O_4-P-CMC/G1.5-3	0.177	0.067	0.041	0.030	0.023	0.019	0.016	0.014	0.012	0.011	0.010

Table S6. The thermodynamic parameters for adsorption of U(VI) onto Fe_3O_4-CMC/G1.5 and Fe_3O_4-P-CMC/G1.5(1-3)

Adsorbents	ΔH°	ΔS°	ΔG° (kJ·mol^{-1})				
	(kJ·mol^{-1})	(J·mol^{-1}·K^{-1})	298. 15(K)	303. 15(K)	308. 15(K)	313. 15(K)	318. 15(K)
Fe_3O_4-CMC/G1.5	27.18	169.47	-21.65	-23.34	-25.04	-26.73	-28.42
Fe_3O_4-P-CMC/G1.5-1	20.11	151.81	-23.63	-25.15	-26.67	-28.19	-29.71
Fe_3O_4-P-CMC/G1.5-2	14.71	124.63	-50.63	-51.88	-53.12	-54.37	-55.62
Fe_3O_4-P-CMC/G1.5-3	19.31	141.03	-21.32	-22.73	-24.14	-25.55	-26.96

Table S7. Distribution coefficient and selectivity coefficients of Fe_3O_4-CMC/G1.5 and Fe_3O_4-P-CMC/G1.5-2

Ions	K_d (mL·g^{-1})		$S_{U(VI)/M(x)}$		Sr
	Fe_3O_4-CMC/G1.5	Fe_3O_4-P-CMC/G1.5-2	Fe_3O_4-CMC/G1.5	Fe_3O_4-P-CMC/G1.5-2	
Zn	0.74	0.50	11.72	21.90	1.87
Ni	0.59	0.43	14.66	25.31	1.73
Co	0.60	0.50	14.15	21.90	1.55
Sr	0.74	0.62	11.71	17.66	1.51
Ce	0.76	0.79	11.39	13.76	1.21
Gd	0.53	0.52	16.38	21.18	1.29
La	0.59	0.59	14.66	25.31	1.73
Sm	1.25	1.15	6.90	9.54	1.38
U	8.62	10.97	1	1	1
